# Gaming Your Mental Health: A Narrative Review on Mitigating Symptoms of Depression and Anxiety Using Commercial Video Games

**DOI:** 10.2196/26575

**Published:** 2021-06-16

**Authors:** Magdalena Kowal, Eoin Conroy, Niall Ramsbottom, Tim Smithies, Adam Toth, Mark Campbell

**Affiliations:** 1 Lero, The Science Foundation Ireland Research Centre for Software Physical Education and Sport Sciences Department University of Limerick Castletroy, Limerick Ireland

**Keywords:** commercial video games, mobile phone, clinical, mental health disorders, psychotherapy, pandemic, accessibility, health care

## Abstract

Globally, depression and anxiety are the two most prevalent mental health disorders. They occur both acutely and chronically, with various symptoms commonly expressed subclinically. The treatment gap and stigma associated with such mental health disorders are common issues encountered worldwide. Given the economic and health care service burden of mental illnesses, there is a heightened demand for accessible and cost-effective methods that prevent occurrence of mental health illnesses and facilitate coping with mental health illnesses. This demand has been exacerbated post the advent of the COVID-19 pandemic and the subsequent increase in incidence of mental health disorders. To address these demands, a growing body of research is exploring alternative solutions to traditional mental health treatment methods. Commercial video games have been shown to impart cognitive benefits to those playing regularly (ie, attention control, cognitive flexibility, and information processing). In this paper, we specifically focus on the mental health benefits associated with playing commercial video games to address symptoms of depression and anxiety. In light of the current research, we conclude that commercial video games show great promise as inexpensive, readily accessible, internationally available, effective, and stigma-free resources for the mitigation of some mental health issues in the absence of, or in addition to, traditional therapeutic treatments.

## Introduction

### Mental Health Disorders

Mental health disorders affect more than 14% of the global population [1] and are estimated to become 1 of the 3 major causes of morbidity and mortality by 2030 [2]. The current global pandemic and subsequent periods of economic uncertainty could increase the prevalence of symptoms of mental health disorders, thus increasing the ubiquitous and widespread requirement for mental health treatments [3]. This poses serious consequences for individuals and society by overburdening the current systems in place [4]. Depressive and anxiety disorders are the most prevalent mental health disorders in the general population, with nearly 264 million people (3.4% of global population) and 284 million people adversely affected by depression and anxiety disorders, respectively [1,5]. Depression and anxiety are often experienced simultaneously, with up to 81% of individuals having an anxiety disorder having a depressive disorder too in their lifetime [6]. Depression and anxiety are also associated with chronic physical comorbidities, such as somatoform disorders [7], cancer, stroke, acute coronary syndrome [8], cardiovascular diseases, diabetes, chronic pain, and visual and auditory impairments [9]. Notably, mental health problems are underreported worldwide [10,11]. Due to various types of stigma and beliefs associated with the causes of symptoms, individuals with a known mental disorder often fail to reach out for help [12-14]. Coupled with this, approximately 70% of those requiring treatment have limited access due to insufficient government funding [15]. These aforementioned factors contribute to the mental health *treatment gap* [15] that exists in high-income countries [16] and that is wider in low- to middle-income countries [17]. As of 2020, 63.2% of the world’s population was reported to have access to the internet in 2020 [18], with a vast majority of households owning a computer (ie, 82% of households in the United Kingdom and 93% of households in Finland) [19]. This increase in internet access and computer ownership has facilitated accessibility to video gaming, with approximately 2.7 billion video gamers reported worldwide in 2020. Given the existing mental health care challenges (high costs, long waiting lists, limited technological support, and less alternatives to traditional mental health care), this paper examines recent research on the potential for commercial video games to ameliorate symptoms in the two most prevalent mental health disorders: depression and anxiety. We discuss the increase in prevalence and severity of these disorders globally and the potential benefits of commercial video games to meet some of the current mental health care challenges associated with these two disorders.

### Background

From the advent of the current worldwide COVID-19 pandemic, subsequent social distancing, quarantining, and restrictive measures have been implemented. The increasing incidence of psychological risk factors such as loneliness [20], stress [21], and general poor mental health [22] have been highlighted globally and are a major cause of concern. This has been supported recently by Brooks et al [23], who identified that common psychological responses, such as poor mental health state, fear, posttraumatic symptoms, anxiety symptoms, anxiety-induced insomnia, and confusion, are increasingly occurring because of lockdowns and quarantines. Thus, not only is the development of these symptoms a problem but the risk of relapse and exacerbation is also elevated among such preexisting mental health issues [24]. Moreover, recent findings suggest that these issues are more likely to occur in developed countries [25]. Prolonged durations of isolation were also found to be associated with poor mental health, posttraumatic symptoms [23], and the so-called *lockdown loneliness* [26]. Unsurprisingly, statistics for anxiety and depressive disorders in April-May 2020 reveal that in comparison with the first half of 2019 [27], US adults were 3 times more likely to screen positive for one or both the disorders and 8 times more likely to screen positive for serious mental distress in 2020 (28%) than in 2018 (3.4%) [26]. Furthermore, it seems that young adults, aged between 18 and 44 years, are particularly affected by the pandemic [20,28,29]. In comparison with 2018, 32.4% of young individuals reported feeling downhearted or depressed with levels of satisfaction with life falling by 80% [29]. In addition, young adults were more affected by loneliness [20] and serious mental distress [28] than older age groups in 2020. Research around the world indicates that women are at a higher risk than men and are more prone to psychiatric disorders during the COVID-19 pandemic [3,20,21,23]. Finally, the increase in depressive and anxiety disorders has also been linked to an increased incidence of burnout, specifically due to financial losses, and interruption of professional activities and social life [30].

### The Cost of Mental Health Care Globally

Although significant progress has been made toward developing empirically supported psychological and psychiatric treatments, a significant proportion of people with mental health problems do not receive these treatments [31]. Finding ways to effectively disseminate treatment is imperative. The most commonly implemented treatments for prevalent mental health issues (ie, depression and anxiety) are pharmacological, psychological, or a combination of the two. The monthly cost of these treatments can range drastically from country to country, with costs ranging from approximately US $370 in the United States [32] to between US $108 and US $340 on an average across Europe [33]. The high cost makes access to a broad number of socioeconomic groups across the globe difficult [34-36]. The cost and availability of mental health care could vary greatly among insurance companies, with health insurance policies not covering mental health care in all cases [4]. Finally, waiting lists for state health care around the globe are a major cause of concern. The median wait time in the European Union to receive an initial assessment of psychiatric needs is between 7 and 30 days [37]. For example, Ireland’s waiting list for mental health services exceeds 10,000 individuals, and the corresponding waiting times can exceed 24 months for an initial assessment [38]. With such long waiting periods in public systems, many individuals might be forced to seek options in more expensive private health care settings. However, the cost barrier to private treatment is often a more salient issue. For example, private therapy sessions can cost US $30-$400 per hour in China, US $45-$224 in Canada, US $30-$60 in the United Kingdom, US $77-$132 in France [39], and US $70-$156 in Ireland [40]. With state-provided health care systems already overwhelmed, the cost barrier of private treatment is felt more acutely and contributes to increasing the socioeconomic divide in terms of access to mental health treatment [17]. With the current issues surrounding traditional mental health treatment highlighted above, there is a need for accessible, affordable, and empirically supported alternative treatment options.

### Traditional Mental Health Care Versus Traditional Alternatives and Digital Technology

Traditional mental health care often includes cognitive behavioral therapy, psychosocial or self-care interventions, and the growing use of digital technologies, such as web-based and smartphone-based delivery [41]. Some therapists incorporate board games, such as tabletop role-playing games (RPGs) like *Dungeons and Dragons* [42] or games like *Dixit* [43], into their therapeutic sessions. There is a myriad of evidence for alternatives to traditional mental health care [44-47]. Some specialists include self-care lifestyle interventions (eg, diet, exercise, and meditation) [48,49]. Research documenting interventions with digital technologies has shown that these types of interventions can improve the quality and accessibility of mental health care in high- and low-income countries [50]. A multitude of mental health–focused smartphone apps have been developed to assist with the implementation of these interventions. Despite promising findings in this area, a recent meta-analysis by Lecomte et al [46] noted that to date, only approximately 3% of mental health apps have been thoroughly validated by research. Therefore, authors have called for high-quality research on the efficacy of mental health apps as a stand-alone self-management option and an additional treatment option for mental health issues. Extending the theme of technology, some researchers and clinical practitioners noted the promise of video games tailored to assist with the treatment and management of a wide variety of clinical issues [51]. These games have been found to be successful in improving mental health outcomes in a variety of domains [51-54]. Many bespoke video games (eg, the Pesky gNATs game) seem to be beneficial as alternatives to traditional treatments [55,56]. Nevertheless, only a few of these games are commercially available (ie, *Into the Woods* and *MindLight*), and unfortunately, most of these games cannot be used without the guidance of qualified personnel [57]. Given the dearth of research, lack of randomized controlled trials (RCTs), and meagre quality of literature on bespoke video games to date, Eichenberg and Schott [52] suggest that current findings (although promising) lack generalizability and that further research is required.

## New Perspective: The Case for Commercial Video Games

### Benefits of Commercial Video Games

In contrast to bespoke clinical video games being developed and customized to address clinical issues for a particular cohort or group, commercial video games are generally available video games primarily designed for entertainment and not therapeutic purposes. Commercial video games are widely accessible and have risen from a niche form of entertainment in the 1970s to become a ubiquitous part of modern society [58]. The ubiquity of the commercial video game industry can be attributed to several factors; however, none are more pertinent than both the enjoyment of play and widespread accessibility. The fact that commercial video games can be engaged with on a computer, mobile phone, or gaming console with internet access [58], and are affordable and readily available, ranging from free-to-play games to games worth US $71, they are commonplace in most households worldwide [59]. Initial studies by Granic et al [60] documented various potential benefits of playing commercial video games. Since then, a growing body of recent evidence suggests notable benefits related to the use of commercial video games for socialization [61], cognition [62-64], emotion regulation [65], and mental health [52,66]. With this in mind, this paper provides a state-of-the-art overview of the current research on the benefits of commercial video games specifically for mental health. With recent evidence and increased demands for mental health care, we aim to highlight the potential of commercial video games to be an inexpensive, readily accessible, worldwide, effective, and stigma-free resource for the prevention and mitigation of mental health issues.

### Current Evidence for Commercial Video Games to Support Mental Health

The potential health benefits of commercial video games have been examined in the clinical population. For example, studies have investigated the advantages of using commercial video games among those with depression [67], anxiety [68], schizophrenia [69], cancer [70] and in older adults [71]. Steadman et al [66] demonstrated psychotherapeutic benefits of specific commercial video game genres (as listed by the authors: *first-person shooters and action games*, *RPGs*, *simulation games*, *miscellaneous and sports games*, *strategy*, and *other genres*) and have provided recommendations for mental health professionals (ie, rapport building and social co-operation). More recently, the potential of commercial video games to successfully combat symptoms of depression, anxiety, and stress is highlighted during the COVID-19 pandemic [72]. In the following sections, we present the latest research highlighting the potential for commercial video games to mitigate symptoms associated with the 2 most prevalent mental health disorders in the general population: anxiety and depression (refer to Table 1 for a summary).

**Table 1:** Selected studies of commercial video games and their reported positive mental health outcomes related to depression and anxiety.

Mental health aspect examined	Game or genre	Outcome	Reference
Prosocial behaviors and decreased loneliness	AVG^a^, RPG^b^, multiplayer games, and video game play	AVGs (specifically with multiplayer functions), RPGs, and video game play all showed benefits for socialization among clinical and nonclinical populations	[66,73,74]
Cognition	AVGs, strategy games, exergames, *Boson X* (CVG^c^), and *Rayman* (CVG)	All games identified a range of cognitive improvements (eg, high executive function and visuospatial perception) in clinical and nonclinical populations. In addition, games were found to mitigate symptoms of dyslexia	[66,74,75]
Goal achievement	*Portal 2* (CVG), *Team Fortress 2* (CVG), and RPGs	All games examined showed improvements in goal-setting behavior and motivation to attain said goals	[60]
Positive reappraisal and mood repair	*Portal 2* (CVG), *Mario Kart* (CVG), *Slenderman* (CVG), *Flappy Bird* (CVG), *Tap the Frog* (CVG), and RPGs	Related to goal achievement, all games showed benefits with mood repair, both in time and magnitude	[60,76,77]
Emotional regulation	*Portal 2* (CVG), *Slenderman* (CVG), *Flappy Bird* (CVG), *Tap the Frog* (CVG), RPGs, and video game play	All games facilitated coping with strong emotions and regulating strong emotive experiences	[60,65,73,74,77,78]
Depressive mood	*Candy Crush* (CVG), *Angry Birds* (CVG), *Limbo* (CVG), and casual games	Casual game interventions decreased negative affect by promoting enjoyment, flow states, and motivation	[73,79]
General anxiety	*MindLight* (strategy game), *Max and the Magic Marker* (CVG), *Rayman* (CVG), Nintendo Wii Exergames, and RPGs	Strategy games, CVG, and CVG exergames all showed significant decreases in general measures of anxiety both immediately after play and maintained with continual play	[74,80-83]
Anxiety prevention	*Rayman* (CVG)	*Rayman* reduced general anxiety to the same degree as a strategy game designed to prevent anxiety	[74,82]
State anxiety	*Plants vs. Zombies* (CVG), *Bejeweled II* (CVG), *Peggle* (CVG), *Bookworm Adventures* (CVG), and casual games	Casual games reduced state anxiety by promoting flow states and goal achievement	[68,84]
Trait anxiety	*Bejeweled II* (CVG), *Peggle* (CVG), *Bookworm Adventures* (CVG), and casual games	Casual games reduced trait anxiety by decreasing levels of general anxiety	[68]
Preoperative anxiety	*Angry Birds* (CVG)	*Angry Birds* reduced preoperative anxiety in children older than 36 months, while maintaining reduced levels of anxiety post operation	[85]
COVID-19 anxiety	CVG exergames	Helped combat anxiety related to the pandemic, lockdowns, and social isolation	[86]

^a^AVG: action video game.

^b^RPG: role-playing game.

^c^CVG: commercial video game.

### Benefits of Commercial Video Games for Symptoms of Depression

The two core symptom criteria of depression are either chronic low mood or loss of interest and loss of pleasure (anhedonia). Depression is generally characterized by changes in affect, cognition, neurovegetative functions, and interepisode remissions [87]. Apart from physical manifestations, such as fatigue, insomnia or hypersomnia, weight loss, and digestive issues, individuals with depression can experience a diminished ability to think and concentrate, and experience greater indecisiveness, feelings of worthlessness, and excessive guilt with recurrent thoughts of death or suicide. Existing research has identified critical cognitive processes underlying depression, such as cognitive biases [88], deficits in cognitive control [89], difficulties with disengagement from negative stimuli [90], reduced inhibition [91], and increased rumination [92]. Such research has identified the use of distraction, acceptance, or reappraisal (flexible reframing of stimuli or situations to change their emotional valence or meaning) as effective strategies to fight depressive symptoms [93].

Regarding anhedonia experienced by individuals with depression, recent evidence has shown the utility of video games to evoke positive emotions such as joy and happiness [65], appreciation and competence [94], and social connectedness [73] in individuals. Referring to the latter, the literature shows that weak connectedness, loneliness, and social isolation [95]; a debilitated sense of psychological belonging [96,97]; and internalized stigma [98] are associated with depressive symptoms. Given the abovementioned links and accepting that the pandemic has entailed less movement and social restrictions, commercially available web-based multiplayer games might be a potentially viable tool to connect isolated individuals. For example, researchers have noted the efficacy in games such as *Minecraft* or *Animal Crossing: New Horizons* for social connectedness, fighting loneliness, maintenance of social interaction, and ultimately the alleviation of depressive symptoms [99-101].

An array of research alludes to the possible benefits of RPGs for individuals with depression. RPGs are most commonly recognized by users’ immersion with an avatar or a played role and require achieving goals according to rules within a virtual world [102]. In early work, Steadman [66] noted that RPGs, through self-identification with played characters, could be used by psychotherapists to generate and test schemas on how individuals function. Moreover, it challenged their identified relations and promoted healthy actions. As such, Zayeni et al [74] highlighted that RPGs can serve as a therapeutic tool to challenge and even change ingrained patterns of thinking, promote generation of positive alternatives, and act as a means to question self-schemas among patients undergoing cognitive behavioral treatment. Such benefits are similar to those of the increasingly popular medium of play therapy [103]. Turning to cognitive behavioral outcomes, researchers have shown that games such as *Portal 2* or RPGs (ie, *World of Warcraft*, *Pokémon*, and *Final Fantasy*) can promote goal achievement and reappraisal and facilitate flexibility and efficient emotional regulation as an adaptive tool [60]. In addition, overall improvements in depressive mood with commercial video game use are seen with the use of the Wii racing game *Mario Kart* among adolescents [76]. In addition, this age group seems to benefit in general from gaming, as regular adolescent gamers showed superior emotional regulation abilities than nongamers [78].

In addition, an interesting 6-week EmotivaMente training intervention based on a range of 13 commercial video games has been recently developed and incorporated with school training for adolescents by Carissoli and Villani [77]. Although the study failed to show improvements in emotional intelligence outcomes and the use of adaptive emotion regulation strategies post training, participants showed improvements in cognitive reappraisal and the expression of emotions in relation to the self. Given these findings, it might be beneficial to thoroughly investigate a similar longitudinal intervention in individuals with depression. In addition, a very recent systematic review on casual commercial video games (ie, *Candy Crush* and *Angry Birds*), often played in short sessions on daily commutes, for example, reported that even 30-minute interactions are capable of reducing depressive symptoms and stress [79]. We also know that distraction (ie, reading a book, playing games, or music) serves as an effective mood-regulatory strategy when used in moderation. Some individuals with depression may find it difficult to initiate distraction spontaneously due to increased rumination and inhibition deficits [104]. A recent paper by Kühn et al [75] demonstrated that the fast-paced commercially available action video game *Boson X* not only improved cognitive outcomes but also showed promise in reducing rumination among individuals with depression.

Commercial video games can be helpful in evoking joy, happiness, and positive mood. Research conducted by Kneer et al [105] indicates that due to in-game success and challenge, the game *Team Fortress 2* can elicit positive postgame mood, and these mood benefits are conferred among players of all skill levels. Art therapy, with its use of visual artistic creation to focus on creativity, symbolism of colors or shapes, and the exploration and expression of personal emotions, could also be seen to permeate into commercial video games with positive outcomes for individuals with depression [106,107]. Distinctly designed gaming environments accompanied by tailored soundtracks serve as a safe setting for the exploration and interpretation of emotional states elicited by game graphics. Wolf [2] links these attributes with the gameplay of commercial video game titles, including *Limbo*, *Journey,* or *Flower*, noticing as well that *Limbo* players self-report improved depressive mood outcomes.

### The Benefits of Commercial Video Games for Symptoms of Anxiety

Depression and anxiety are often comorbid disorders [6]. Consequently, exploring the potential of commercial video games to alleviate the symptoms of anxiety is also needed. Anxiety disorders (ie, excessive behavioral disturbances, worry, and fear) can cause significant distress and impairment in social, occupational, and other areas of an individual’s functioning [87]. Separation anxiety, social anxiety, generalized anxiety disorders, anxiety-specific phobias, and panic attacks are some of the subclassifications of anxiety disorders [87]. Anxiety disorders are also associated with difficulty concentrating during work and play, and they can lead to excessive fear of negative evaluation. Different frameworks indicate anxious apprehension and automatic preparatory behavior as key components of anxiety [108,109].

In a recent systematic review, Zayeni et al [74] show that some commercially available video games can be effectively used to address some of the symptoms of anxiety disorders, help treat general symptoms of anxiety (ie, *MindLight* and *Max and the Magic Marker*), reduce measures of social anxiety (ie, *Adventures aboard the S.S. GRIN*), and promote anxiety prevention (ie, *Rayman*). Furthermore, Ohannessian [110] found that web-based video games lowered anxiety levels and increased levels of social connectedness in adolescent boys. Moreover, an intervention by Fish et al [84] compared a prescribed 30- to 45-minute session of *Plants vs. Zombies*, a tower defense game, 4 times per week with a traditional pharmacological selective serotonin reuptake inhibitors treatment for individuals with depression and comorbid anxiety. This study revealed a greater reduction in state anxiety severity (State-Trait Anxiety Inventory questionnaire) [111] in the gaming group than in the medication group. Similarly, prescribed commercial casual video games have been shown to significantly decrease both state and trait anxiety in patients diagnosed with depression and comorbid anxiety [68].

Use of commercial video games also appears to be beneficial in facilitating anxiety management in a clinical setting [112], showing effectiveness in improving the management of preoperative anxiety in pediatric patients [85]. Reduced levels of anxiety were also evident in patients with Parkinson disease who participated in an exergames intervention using the Nintendo Wii console [80]. Furthermore, commercially available exergames, which combine physical exercise and gaming, show promise for their anxiolytic effects. As presented in the systematic review and meta-analysis by Viana et al [81], it has been reported that these games provide similar results and high adherence when compared with rehabilitation or usual care, additionally bringing fun and enjoyment to anxious individuals using them. More recently, it has also been suggested that the use of commercial exergames might serve as a strategy to cope with anxiety during quarantine [86].

When comparing bespoke video games with commercially available video games, an RCT presented equal improvements in anxiety levels between groups of adolescents who played either the bespoke biofeedback game *Dojo* or the commercially available *Rayman 2: The Great Escape* 6 times over 3 weeks [82]. Another RCT study exhibited similar effects for 2 games believed to optimize emotional intensity, capturing the same motivation and engagement levels of players [83]. Initially designed for therapeutic purposes and now commercially available, *MindLight* was compared with another commercially available game, *Max and the Magic Marker*, sharing similar action mechanics. Both these games demonstrated prevention of elevated anxiety in children. The fact that commercial video games appear to be equally effective as bespoke video games in reducing symptoms of anxiety is extremely promising when considering their overall therapeutic potential, cost-effectiveness, and ease of use.

## The Future of Commercial Video Games

The advent of virtual reality (VR) video games at home has begun, and with it, researchers and video game companies are beginning to find new solutions to address mental health support through VR gaming [113]. A recently published study by Pallavicini and Pepe [114] showed significant decreases in state anxiety and negative emotions, accompanied by increased levels of positive emotions, for young adults who played the commercially available VR games *Fruit Ninja VR* and *Audioshield*. Commercially available VR video games have great potential and are well suited for the implementation of cognitive behavioral techniques for the treatment of depressive and anxiety disorders. Given the immersive nature of VR technology and the controllability of the virtual environment, it could be particularly well suited for use in exposure therapy. Exposure therapy is often prescribed to individuals with anxiety disorders [115]. Although research is still preliminary, VR commercial video games show great promise for treating and coping with symptoms of mental health issues; however, further scientific research is required to validate these initial promising findings.

Mental health treatment options often emphasize the role of active involvement in the therapeutic process [116,117], which often results in reduced anxiety levels and stress-related symptoms. Pine et al [79] suggested that providing an option to choose among many casual video game options might be important in promoting autonomy and, thus, may increase the positive outcomes of interventions using commercial video games. Given the large variety (>1 million) [118] of existing commercial video games and varied means of their delivery (PCs, phones, tablets, consoles, and VR) on the market, there is a great opportunity for therapists and researchers to examine this wide range of commercially available games within the broad context of mental health. Therefore, further research should investigate the theoretical underpinnings of the effect of video game play on mitigating mental health symptoms. Although a growing body of research has examined the influence of commercial video gaming on mental health in diverse settings, the emphasis of future work should be on various populations and across different demographics. With the increased prevalence of mental health disorders, each age group could find a selection of commercial video games that are well suited for their needs, preferences, and age-related demands.

Some video gaming companies (ie, *thatgamecompany* and *Glitchers*) have noted the potential of video games to improve mental health outcomes and have based their game design on psychological theories and art and music therapies to favor the potential mental health benefits of users. Music engages several brain structures and neurotransmitters related to reward, motivation, pleasure, and stress [119], with music therapy purported to effectively reduce symptoms of depression and anxiety [120-122] and health promotion [123]. Many musical rhythm video games or games with singing inputs exist already (eg, *Guitar Hero, Let’s Sing,* and *Beat Saber*). However, research examining singing games [124] and musical games [125,126] in general or the possible influence of music within video games on mental health benefits to date [127] is still in its infancy. Given that musical elements are essential to contemporary commercial video games (soundtracks), close collaborations between game developers and musicians already exist [128]. We argue that research could further this avenue of future multidisciplinary collaboration by considering and exploring the role played by such auditory and musical elements in improving mental health outcomes in gaming and digital contexts.

## Conclusions

Given the aforementioned research on issues faced by mental health services and exacerbated by the recent worldwide pandemic, we are currently facing a serious threat to mental health globally. With many patients on waiting lists or unable to receive support, we refer to the purpose of this paper, which was to present the potential for commercial video games to ameliorate the symptoms of the 2 most prevalent mental health disorders: depression and anxiety. Overall, we have demonstrated the current evidence wherein commercial video games serve as a useful corollary and easily accessible tool for decreasing the severity of symptoms of depression and anxiety. Commercial video games are usually either freely available or available at a one-time, relatively low cost. In addition, commercial video games possess many important features needed (eg, schemas, controlled in-game scenarios related to specific mental health issues, design, user engagement, and immersive state of *flow*) to make them effective as a preventative tool or for supplementing traditional therapies in the treatment of anxiety and depressive disorders. Compared with most clinical treatments (eg, pharmacological and psychological), commercial video games do not require in-person supervision, can be accessed remotely, are low cost (often providing no financial burden), are readily available, are easy to implement, are portable, and carry a greatly reduced risk of adverse side effects. Moreover, gaming, as a form of entertainment, is already popular among those most affected by mental health issues these days, as the majority of gamers are 18 years or older (79% of gamers), with 41% being female [58]. An average gamer is 34 years old, with individuals aged between 18 and 34 years comprising 38% of video gamers, followed by individuals aged between 34 and 54 years (26%) [58].

In conclusion, the overall accessibility and pervasiveness of commercial video games within modern society positions them as an invaluable means of reaching individuals with mental health disorders, irrespective of age and sex, and individuals with limited access to mental health care, particularly relevant during the current pandemic. With mounting scientific evidence in support of the efficacy of commercial video games for improving mental health outcomes, commercial video games should be considered as a potential alternative for the improvement of various aspects of mental health globally.

## Abbreviations

RCT: randomized controlled trial
RPG: role-playing game
VR: virtual reality
